# Spontaneously Arrested Bilateral Primary Congenital Glaucoma: A Case Report From Ethiopia

**DOI:** 10.4314/ejhs.v32i2.27

**Published:** 2022-03

**Authors:** Kalkidan S Alayu, Menen A Shibeshi, Abiye M Alemu

**Affiliations:** 1 Department of Ophthalmology, School of Medicine, College of Health Sciences, Addis Ababa University, Ethiopia

**Keywords:** Megalocornea, Primary congenital glaucoma, spontaneously arrested primary congenital glaucoma, Subluxated cataract

## Abstract

**Background:**

Primary congenital glaucoma is potentially blinding condition characterized by elevated intraocular pressure and optic disc cupping. It is typically bilateral and usually manifest in the first year of life. Spontaneously arrested primary congenital glaucoma can occur, but it is very rare.

**Case Report:**

A 32-year-old male patient from North Shewa presented to the department of ophthalmology, Menelik II Hospital with deterioration of vision. On examination he had large corneas with horizontal diameter of 14 mm, increased axial length, faint corneal stromal opacity and Haab's striae of both eyes. Anterior chamber angles were wide open. His intraocular pressure, optic nerve head appearance and visual field in both eyes were normal. He had subluxated dense cataract of the right eye.

**Conclusion:**

Late presentation with sequelae of primary congenital glaucoma without optic neuropathy is possible. Regular follow-up of spontaneously arrested congenital glaucoma and scleral fixation of intraocular lens is recommended.

## Introduction

Primary congenital glaucoma (PCG) is a rare eye disease which occurs at birth or early childhood. The incidence of PCG varies in different populations. The disease is bilateral most of the time, but in 25–30% of the cases may be unilateral. Most of the cases (65%) of them are boys (1).

The Childhood Glaucoma Research Network has standardized and expanded classification of PCG as neonatal, infantile, late onset and spontaneously arrested cases with normal Intraocular pressure (IOP) but typical signs of PCG (2). The signs and symptoms of PCG are corneal enlargement, corneal edema, Haab's striae, refractive error and amblyopia. Optic nerve damage resulting in vision loss and sequelae like Cataract, lens dislocation and retinal detachment has been mentioned (3).

Only few cases of spontaneously regressed glaucoma have been reported so far. We report a bilateral spontaneously arrested primary congenital glaucoma in an Ethiopian man presented with subluxated cataract lenses.

## Case Report

A 32-year-old man from North Shewa, Central part of Ethiopia, presented to Menelik II Hospital in June 2019 with chief compliant of reduction of vision of 2 year duration. The reduction of vision had been since childhood but worsened in the past 2 years. The low vision was worse at distance and he has learned that his eyes were large since childhood. Family history was unremarkable and there was no consanguinity of the parents.

**Figure 1:**
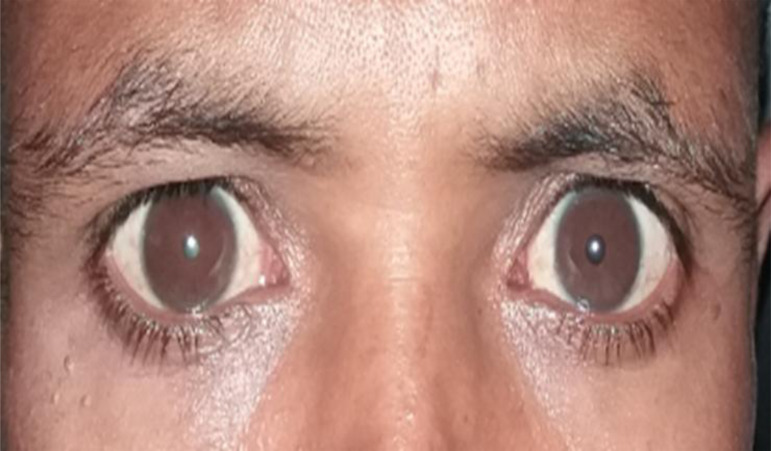
Megalocornea of both eyes

**Figure 2:**
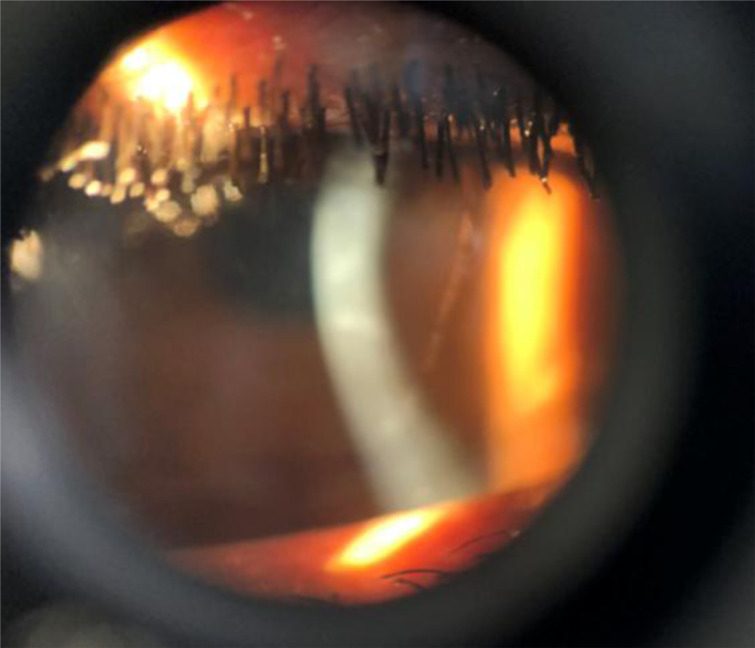
Corneal stromal opacity and Haab's striae of left eye

Physical examination showed that best corrected visual acuity (BCVA) of counting finger at one and three meters in the right and left eyes respectively. Average repeated IOP measured by I- care tonometer was 12 mmHg for right eye and 11 mmHg in the left eye. Pupils were equal in size and reactive to light, there was no relative afferent pupillary defect. Horizontal corneal diameter was 14mm (Figure 1) in both eyes with stromal opacities and Haab's striae (Figure 2). Anterior chambers were deep and gonioscopy revealed wide open angles in both eyes. There was iridodonesis, phacodonesis, as well as superiorly and nasally subluxated cataract lenses in both eyes. The cataract in the right eye was denser.

**Figure 3:**
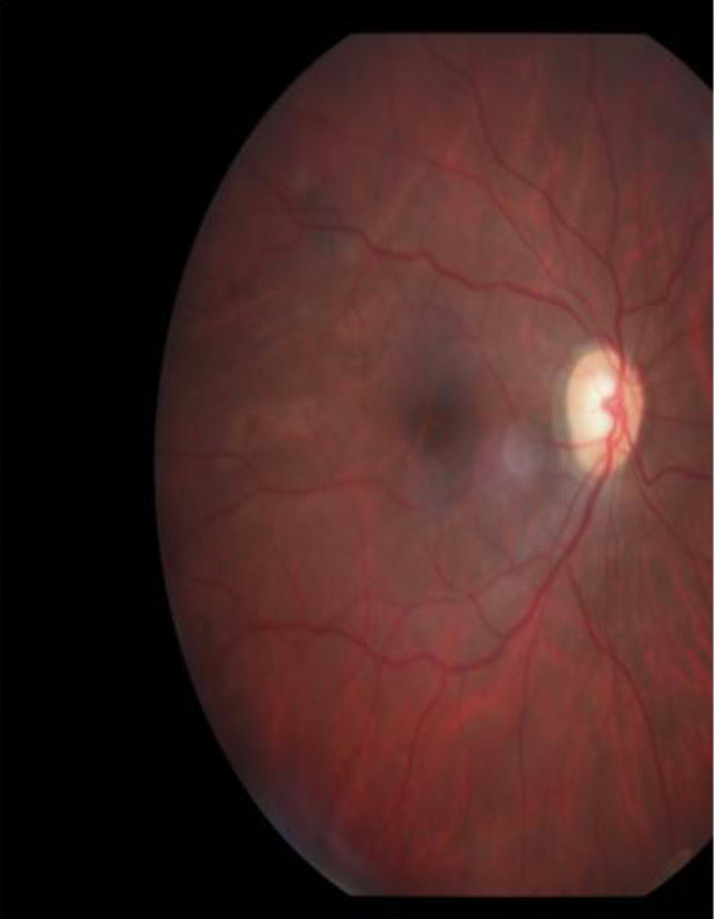
Normal appearance of optic nerve head of right eye (after cataract surgery)

Optic nerve head evaluation by 90D lens showed pink rim with vertical cup- to- disc ratio of 0.6 in both eyes with no nerve fiber layer loss (Figure 3). Visual field examination was normal in both eyes (right eye examination was done after cataract surgery). Central corneal thickness was 466um in the right eye and 467um for the left eye. Axial length was 30.3mm and 29.9mm for right and left eyes respectively.

The diagnosis of subluxated cataract of both eyes, but denser on right and spontaneously arrested primary congenital glaucoma was reached. After counseling and discussion on prognosis with the patient, manual small incision cataract surgery was done for the right eye. On 2^nd^ post-operative week, the right eye BCVA was 6/36 and poor vision was explained by amblyopia. After one month the IOP was normal, but intraocular lens (IOL) was dislocated and BCVA decreased to 6/60.

## Discussion

Our case had a megalocornea, Haab's striae, normal IOP, pink discs with vertical-cup-to-disc ratio of about 0.6, increased axial length, gonioscopic finding of wide open angle and subluxated cataract lenses in both eyes. He presented with deterioration of vision of both eyes. His low vision since childhood could be explained by amblyopia and refractive errors. Finally he had developed cataract that worsened his vision and obliged him to come to Hospital. Megalocornea, increased axial length, Haab's striae, normal IOP and optic disc findings seen in our patient were consistent with the cases described for spontaneously arrested primary congenital glaucoma in the literature (4). Cause of spontaneous resolution is not known, but continued post-natal development has been suggested (5).

Complications can follow standard cataract surgery on patients with megalocornea and zonular weakness. Our patient's IOL was dislocated despite proper placement in the capsular bag, and scleral fixation is recommended.
